# Association between prognostic nutritional index and 28-day mortality in patients with sepsis-associated acute respiratory distress syndrome

**DOI:** 10.3389/fnut.2026.1867675

**Published:** 2026-07-07

**Authors:** Lina Zhao, Peng Li, Shuoyan Dong, Jiajia Tian, Qian Cui, Yuehao Shen, Haiying Liu, Xuguang Li, Yun Li, Keliang Xie

**Affiliations:** 1 Department of Critical Care Medicine, Tianjin Medical University General Hospital, Tianjin, China; 2 Department of Anaesthesiology, Yidu Central Hospital, Weifang, China; 3 Department of Anesthesiology, Tianjin Institute of Anesthesiology, Tianjin Medical University General Hospital, Tianjin, China; 4 Department of Endocrinology, Yidu Central Hospital, Weifang, China; 5 Department of Respiratory and Critical Care Medicine, The First Affiliated Hospital of Baotou Medical College, Baotou, China; 6 Department of Anesthesiology, The Second Hospital of Tianjin Medical University, Tianjin, China

**Keywords:** 28-day mortality, ARDS, ICU, PNI, sepsis

## Abstract

**Background and Aims:**

Despite advances in critical care, sepsis-associated acute respiratory distress syndrome (SA-ARDS) continues to impose a substantial clinical burden, characterized by persistently high incidence and mortality. This study aimed to investigate the association between the Prognostic Nutritional Index (PNI) and 28-day mortality in SA-ARDS patients.

**Methods:**

In this retrospective cohort study, 1,022 adult ICU patients with SA-ARDS (2020–2025) were analyzed. The primary outcome of this study was the 28-day mortality rate in patients with SA-ARDS. Multivariate logistic regression, propensity score matching (PSM), inverse probability weighting (IPW), doubly robust estimation, generalized additive models (GAM), Kaplan–Meier (KM) survival analysis, and receiver operating characteristic (ROC) curve were used to investigate the relationship between PNI and 28-day mortality in patients with SA-ARDS.

**Results:**

All statistical models consistently identified PNI as an independent protective factor for SA-ARDS prognosis (multivariate logistic regression: OR 0.41, 95% CI 0.31–0.51; PSM: OR 0.52, 0.45–0.57; IPW: OR 0.50, 0.44–0.57; doubly robust estimation: OR 0.03, 0.02–0.06). The GAM revealed a notable threshold-dependent association between PNI and 28-day mortality in SA-ARDS patients, with an optimal cutoff value of 41. Patients with PNI ≥ 41 had a markedly reduced mortality risk (*p* < 0.001). ROC comparison via DeLong test revealed heterogeneous performance of standalone PNI across cohorts: PNI outperformed both SOFA and APACHE II in the TMUGH cohort, showed no statistical difference from the two scores in MIMIC-IV, and was superior to APACHE II but comparable to SOFA in eICU. Notably, the combined model integrating PNI with SOFA and APACHE II achieved significantly better predictive ability than any single indicator across all three cohorts.

**Conclusion:**

The PNI is an independent predictor of 28-day mortality in SA-ARDS patients. Although the predictive efficacy of standalone PNI varied across different cohorts, combining PNI with conventional SOFA and APACHE II scores steadily improved prognostic performance. As a simple laboratory-derived indicator, PNI can act as a valuable auxiliary marker to optimize risk stratification for SA-ARDS patients. Further prospective studies are required to verify its clinical utility.

## 1 Introduction

Sepsis, a prevalent critical illness in clinical practice, is defined as life-threatening organ dysfunction caused by a dysregulated host response to infection and accompanied by multiple organ failure, immune dysregulation, and high mortality rates (1). ARDS, a life-threatening complication of sepsis, is a critical syndrome frequently encountered in the intensive care unit (ICU). It is characterized by diffuse alveolar damage, disruption of the alveolar-capillary barrier and pulmonary edema (2). Evidence from clinical studies demonstrates that sepsis contributes to the pathogenesis of 40% of ARDS cases (3). Despite recent advances in the understanding and management of ARDS, the mortality rate remains high at 35–40% (4). Therefore, identifying early prognostic predictors for ARDS is crucial for assessing disease severity and facilitating early individualized interventions to reduce mortality.

The inflammatory response plays a pivotal role in the pathogenesis of ARDS and significantly influences patient outcomes (5, 6). Lymphocytes are closely associated with inflammatory reactions, with studies demonstrating that septic patients exhibit significantly lower absolute lymphocyte counts than healthy individuals, and non-survivors have lower counts than survivors—findings that underscore the link between lymphocyte levels and sepsis-related morbidity and mortality (7). Albumin, on the other hand, suppresses proinflammatory mediators and improves oxygenation in ARDS patients (8, 9). It has also been shown to reduce mortality risk in critically ill patients (10–12). In summary, both lymphocytes and albumin may serve as significant prognostic biomarkers in critically ill patients. The Prognostic Nutritional Index (PNI), a composite indicator combining serum albumin and lymphocyte counts, reflects not only a patient’s nutritional reserves and immune status but also correlates with systemic inflammatory response and oxidative stress levels (13, 14)—key components of ARDS pathophysiology.

However, the relationship between PNI and mortality in patients with SA-ARDS remains unclear. In this study, we aim to examine the association between PNI and 28-day mortality of patients with SA-ARDS.

## 2 Materials and methods

### 2.1 Data description

This retrospective multicenter cohort study analyzed data from three independent sources. Data on SA-ARDS patients admitted to the ICU between January 2020 and November 2025 were extracted from the Tianjin Medical University General Hospital (TMUGH) database. External validation utilized the Medical Information Mart for Intensive Care IV (MIMIC-IV v2.2) and eICU Collaborative Research Database (eICU-CRD v2.0). The study team has completed the Collaborative Institutional Training Initiative Examination and obtained certification. The study protocol received approval from the Institutional Review Board of Tianjin Medical University General Hospital (IRB2025-YX-173-01). Both MIMIC-IV and eICU-CRD datasets were accessed under approved protocols from the Beth Israel Deaconess Medical Center (2001-P001699/14) and Massachusetts Institute of Technology (No. 0403000206), following complete de-identification of protected health information. These databases contain demographic information, treatment records, infection sites, microbiological results, vital signs, laboratory parameters, comorbidities, Sequential Organ Failure Assessment (SOFA) scores and other clinical data.

### 2.2 Patients selection

We included all the patients in these databases who met the following criteria: (1) Diagnosed in accordance with the Sepsis-3 criteria (1), (2) ARDS defined according to the Berlin criteria (15), (3) Age ≥18 years, (4) ICU hospitalization duration ≥ 24 h. Exclusion criteria included: (1) congestive heart failure, cardiogenic pulmonary edema, extensive atelectasis, massive pleural effusion, and massive pulmonary embolism, (2) those not receiving mechanical ventilation, and (3) cases with missing critical parameters (albumin, lymphocyte).

### 2.3 Data extraction

The primary outcome was 28-day all-cause mortality. Oxygen exposure indices were operationalized using median values of Saturation of peripheral Oxygen (SpO_2_), arterial oxygen partial pressure (PaO_2_), Fraction of Inspired Oxygen (FiO_2_), respiratory rate (RR), and positive end-expiratory pressure (PEEP) during invasive mechanical ventilation. Baseline data included demographic parameters (age, sex), comorbidity profiles, infection characteristics (site of infection, microbiological types), critical care interventions (renal replacement therapy, vasopressor/inotrope utilization, mechanical ventilation duration), and ICU length of stay. Physiological severity was quantified using SOFA, Acute Physiology and Chronic Health Evaluation II (APACHE II) scores within 24 h of ICU admission. The median vital signs and laboratory test results of the patients were also collected during hospitalization.

### 2.4 Statistical analysis

All statistical analyses were performed using R software. Continuous variables were assessed for normality using the Kolmogorov–Smirnov test and expressed as mean ± standard deviation for normally distributed data or median (interquartile range) for non-normally distributed data, while categorical variables were reported as numbers (percentages). Baseline characteristics were compared using one-way ANOVA for continuous variables and Pearson’s chi-square test for categorical variables. Generalized additive model (GAM) was used to explore the threshold effect between PNI and 28-day mortality in patients with SA-ARDS. Multivariable logistic regression, propensity score matching (PSM), inverse probability weighting (IPW), and doubly robust estimation were applied to identify the association between PNI and 28-day mortality in patients with SA-ARDS. The 17 clinically relevant variables included in the 1:1 PSM model were as follows: *Acinetobacter baumannii* infection, *Klebsiella pneumoniae* infection, vasopressor use, continuous renal replacement therapy (CRRT), prothrombin time, international normalized ratio, aspartate aminotransferase, activated partial thromboplastin time, blood urea nitrogen, creatine kinase-MB, creatine kinase, creatinine, C-reactive protein, procalcitonin, SOFA score, platelet count (PLT) and lactate (Lac). After matching, the standardized mean differences of all variables were less than 0.1, indicating good baseline balance between the two groups. The detailed balance results are shown in Supplementary material 3. External validation was conducted through Kaplan–Meier survival analysis with log-rank test in MIMIC-IV and eICU databases using the predetermined PNI cutoff. The discriminative ability of PNI, SOFA, APACHE II, and their combined model was assessed using receiver operating characteristic (ROC) curve analysis. The DeLong test was applied to perform pairwise comparisons of ROC curves to statistically evaluate the differences in predictive performance among different models. The correlation between PNI levels and SA-ARDS severity was assessed using box-and-whisker plots. *p*-value < 0.05 was considered statistically significant.

## 3 Results

### 3.1 Baseline demographic and clinical characteristics

According to the inclusion and exclusion criteria, a total of 1,022 SA-ARDS patients were enrolled from the TMUGH database, and 1929 SA-ARDS patients were included from the MIMIC-IV and eICU databases (Supplementary material 1). And then we divided the patients into two groups: the 28-day survivors and non-survivors. The comparative analysis of baseline characteristics between 28-day survivors and non-survivors revealed significant clinical disparities (Table 1 and Supplementary material 2). Non-survivors demonstrated markedly impaired respiratory function, with arterial blood gas analysis showing substantially lower oxygen saturation (SpO₂; *p* < 0.001), arterial oxygen partial pressure (PaO₂; *p* < 0.001), and PaO₂/FiO₂ ratio (*p* < 0.001), accompanied by higher requirements for fractional inspired oxygen (FiO₂; *p* < 0.001) and positive end-expiratory pressure (PEEP; *p* < 0.001). For non-survivors the hemodynamic instability was evident through reduced systolic (*p* < 0.001) and diastolic blood pressure (*p* < 0.01), combined with increased vasopressor dependence (*p* < 0.001) and utilization of continuous renal replacement therapy (CRRT; *p* < 0.001). Microbiological analysis identified a higher prevalence of *Acinetobacter baumannii* (*p* < 0.01) and *Klebsiella pneumoniae*. (*p* < 0.05) infections in non-survivors. Also, the Multi-organ dysfunction was more pronounced manifested by elevated hepatic enzymes (ALT, *p* < 0.001; AST, *p* < 0.001), hyperbilirubinemia (*p* < 0.001), biomarkers of myocardial injury (CK, *p* < 0.001; CK-MB, *p* < 0.01), and impaired renal function (creatinine, *p* < 0.001; blood urea nitrogen, *p* < 0.001). Coagulation abnormalities featured paradoxical thrombocytosis (*p* < 0.05) alongside prolonged international normalized ratio (INR; *p* < 0.001) and activated partial thromboplastin time (APTT; *p* < 0.01). Systemic inflammation was more severe, as evidenced by elevated C-reactive protein (CRP; *p* < 0.001) and procalcitonin (PCT; *p* < 0.001). Non-survivors had significantly higher lactate levels (*p* < 0.001), indicating impaired peripheral perfusion. Both disease severity scores (SOFA and APACHE II) were significantly higher among non-survivors (both *p* < 0.001).

**Table 1:** Baseline characteristics of sepsis-associated ARDS patients.

Variable	Original cohort			Match cohort		
	Survival group (*n* = 587)	Non-survival group (*n* = 435)	*p*	Survival group (*n* = 394)	Non-survival group (*n* = 394)	*p*
Baseline variables						
Age (years), (median [IQR])	68.00 [55.00, 75.00]	67.00 [55.00, 75.00]	0.429	67.50 [55.00, 75.00]	67.00 [55.00, 75.00]	0.737
Gender, F (%)	240 (40.9)	153 (35.2)	0.073	163 (41.4)	154 (39.1)	0.561
Respiratory-related indicators (median [IQR])						
SpO_2_ (%)	97.20 [95.30, 98.10]	93.80 [91.95, 95.85]	< 0.001	97.20 [95.30, 98.10]	92.80 [90.10, 94.10]	< 0.001
FiO_2_ (%)	60 [50, 60]	70 [50, 80]	< 0.001	60 [50, 60]	80 [70, 90]	< 0.001
PaO_2_ (mmHg)	94.20 [88.35, 107.10]	83.30 [75.90, 90.45]	< 0.001	93.90 [88.43, 107.10]	79.60 [72.00, 87.20]	< 0.001
PaO_2_/FiO_2_ (mmHg)	171.00[154.20, 188.25]	127.80[112.30, 172.20]	< 0.001	169.15 [154.90, 186.20]	113.00 [103.20, 126.10]	< 0.001
Duration of MV(day)	8.00 [3.77, 18.00]	8.00 [3.00, 13.13]	0.004	9.61 [5.00, 20.00]	4.85 [2.00, 10.00]	< 0.001
TV.kg. IBW (ml/kg)	7.69 [7.06, 8.66]	7.58 [6.85, 8.22]	0.001	7.69 [7.05, 8.57]	7.58 [6.92, 8.33]	0.077
PEEP (mmHg)	7.20 [6.60, 7.60]	8.00 [7.70, 8.30]	< 0.001	7.10 [6.50, 7.60]	8.00 [7.90, 8.50]	< 0.001
Treatment, *n* (%)						
Muscle Relaxant (%)	17 (2.9)	14 (3.2)	0.91	15(3.8)	12(3.0)	0.695
CRRT (%)	172 (29.3)	197 (45.3)	< 0.001	149(37.8)	140(35.5)	0.554
Prone Ventilation (%)	20 (3.4)	19 (4.4)	0.53	17(4.3)	12(3.0)	0.449
Vasoactive (%)	346 (58.9)	295 (67.8)	0.005	252(64.0)	256(65.0)	0.823
Hormone (%)	122 (20.8)	86 (19.8)	0.749	81(20.6)	62(15.7)	0.096
Laboratory parameters, (median [IQR])						
Albumin(g/L)	31.00 [29.00, 32.00]	31.00 [27.50, 33.00]	0.049	37.00 [33.25, 39.00]	31.00 [30.00, 31.00]	< 0.001
Lymphocytes( × 10^9^/L)	2.40 [1.96, 2.80]	0.80 [0.40, 1.50]	< 0.001	2.44 [2.11, 2.83]	0.86 [0.40, 1.50]	< 0.001
PNI	49.25 [45.77, 52.42]	34.00 [31.73, 38.00]	< 0.001	49.20 [45.80, 52.50]	35.00 [32.10, 38.35]	< 0.001

SpO2, saturation of peripheral oxygen; PaO2, partial pressure of arterial oxygen; FiO2, fraction of inspired oxygen; PEEP: positive end-expiratory pressure; PNI, prognostic nutritional index (10 × albumin [g/dL] + 5 × lymphocyte count [×109/L]); CRRT, continuous renal replacement therapy; MV, mechanical ventilation; TV.kg. IBW: tidal volume per kilogram of ideal body weight. Statistical significance was defined as *p* < 0.05.

To address potential confounding factors, 1:1 propensity score matching was implemented using 17 clinically relevant variables (Supplementary material 3). The matched cohorts demonstrated adequate balance across all parameters (standardized mean differences <0.1; Supplementary material 3), with PNI remaining significantly elevated in survivors compared to non-survivors (*p* < 0.001).

### 3.2 Threshold effect of PNI on 28-day mortality in patients with SA-ARDS

**Figure 1:**
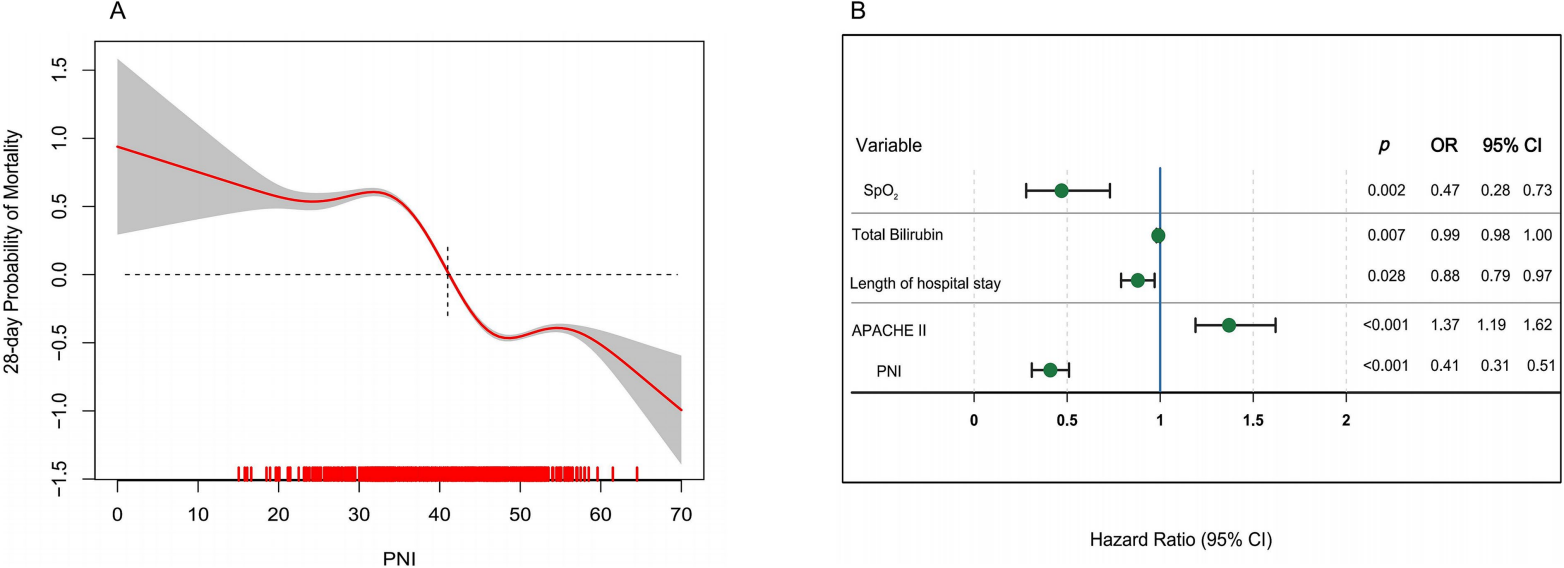
Association between PNI and 28-day mortality in patients with SA-ARDS. **(A)** Generalized additive model curve showing the threshold effect of PNI on 28-day mortality; the optimal cutoff value was determined as 41. **(B)** Forest plot of independent predictors derived from multivariate logistic regression analysis. PNI, Prognostic Nutritional Index; SA-ARDS, sepsis-associated acute respiratory distress syndrome; OR, odds ratio; CI, confidence interval; SpO_2_, peripheral oxygen saturation.

The GAM identified a prominent threshold effect between PNI and 28-day mortality in SA-ARDS patients (Figure 1A). A clear inflection point was observed at the PNI value of 41, above which patients exhibited a significantly lower 28-day mortality risk (*p* < 0.001) (Figure 1A).

### 3.3 Multiple models analysis for the relationship between PNI and 28-day mortality in patients with SA-ARDS

Multiple analytical approaches consistently identified PNI as a protective factor against 28-day mortality in SA-ARDS patients: multivariable logistic regression (OR 0.41; 95% CI 0.31–0.51), PSM (OR 0.52; 95% CI 0.45–0.57), IPW (OR 0.50; 95% CI 0.44–0.57), and doubly robust estimation (OR 0.03; 95% CI 0.02–0.06) (Table 2). The extremely low OR from doubly robust estimation stems from its comprehensive dual-confounder adjustment strategy, which strengthens the protective effect of PNI. The significant predictors identified in the multivariate logistic regression analysis were graphically summarized in Figure 1B, with complete variable analyses provided in Supplementary material 4.

**Table 2:** Multiple models analysis between PNI and 28-day mortality in sepsis-associated ARDS patients.

Models	OR	95%CI		*p*
		Lower	Upper	
Multivariate logistic analysis*	0.41	0.31	0.51	< 0.001
Propensity score matching*	0.52	0.45	0.57	< 0.001
Inverse probability weighting*	0.50	0.44	0.57	< 0.001
Doubly robust with all covariates #	0.03	0.02	0.06	< 0.001

*PNI for continuous variables; The doubly robust method involves converting the continuous PNI variable into a binary categorical variable based on the cut-off value for analysis; OR, Odds Ratio; CI, Confidence Interval; *p* < 0.05, statistically significant; PNI, Prognostic Nutritional Index = 10 × Albumin(g/dL) + 5 × Lymphocyte(× 10^9^/L).

### 3.4 External validation in the MIMIC - IV and eICU cohorts

**Figure 2:**
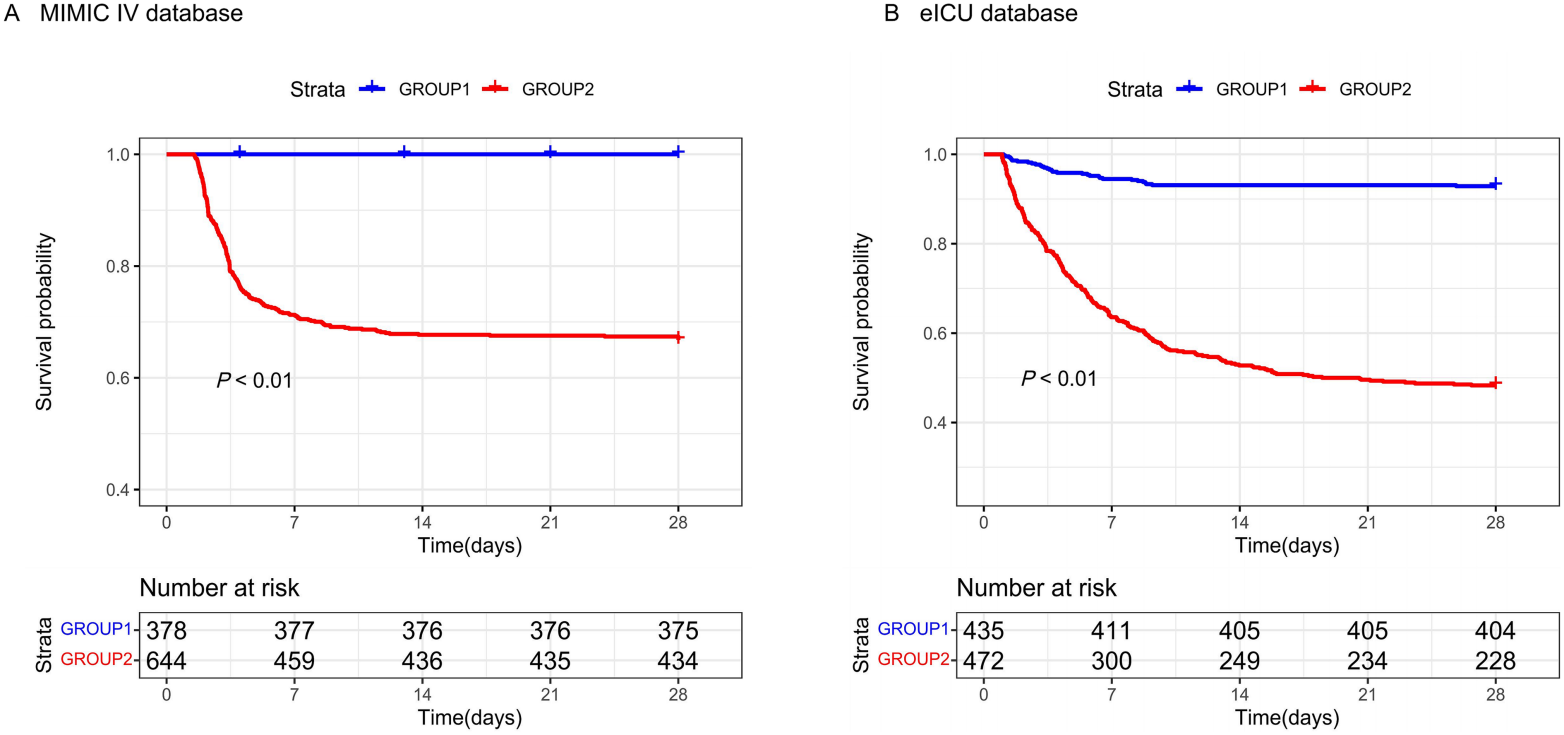
Kaplan–Meier survival curves for 28-day mortality stratified by PNI level. Group 1: PNI < 41, GROUP2: PNI ≥ 41; **(A)** Survival analysis in the MIMIC-IV cohort (*p* < 0.001). **(B)** Survival analysis in the eICU cohort (*p* < 0.001). PNI, Prognostic Nutritional Index; SA-ARDS, sepsis-associated acute respiratory distress syndrome; MIMIC-IV, Medical Information Mart for Intensive Care IV.

External validation using the predefined PNI cutoff of 41 was performed through Kaplan–Meier analysis in two independent critical care databases (MIMIC - IV and eICU). As shown in Figure 2, both cohorts demonstrated that patients in low-PNI (< 41) group had a significantly higher mortality rate than those in high-PNI (≧ 41) group (*p* < 0.0001).

### 3.5 Prognostic value of PNI for 28-day mortality in SA-ARDS

#### 3.5.1 Prognostic value of PNI for 28-day mortality in SA-ARDS

**Figure 3:**
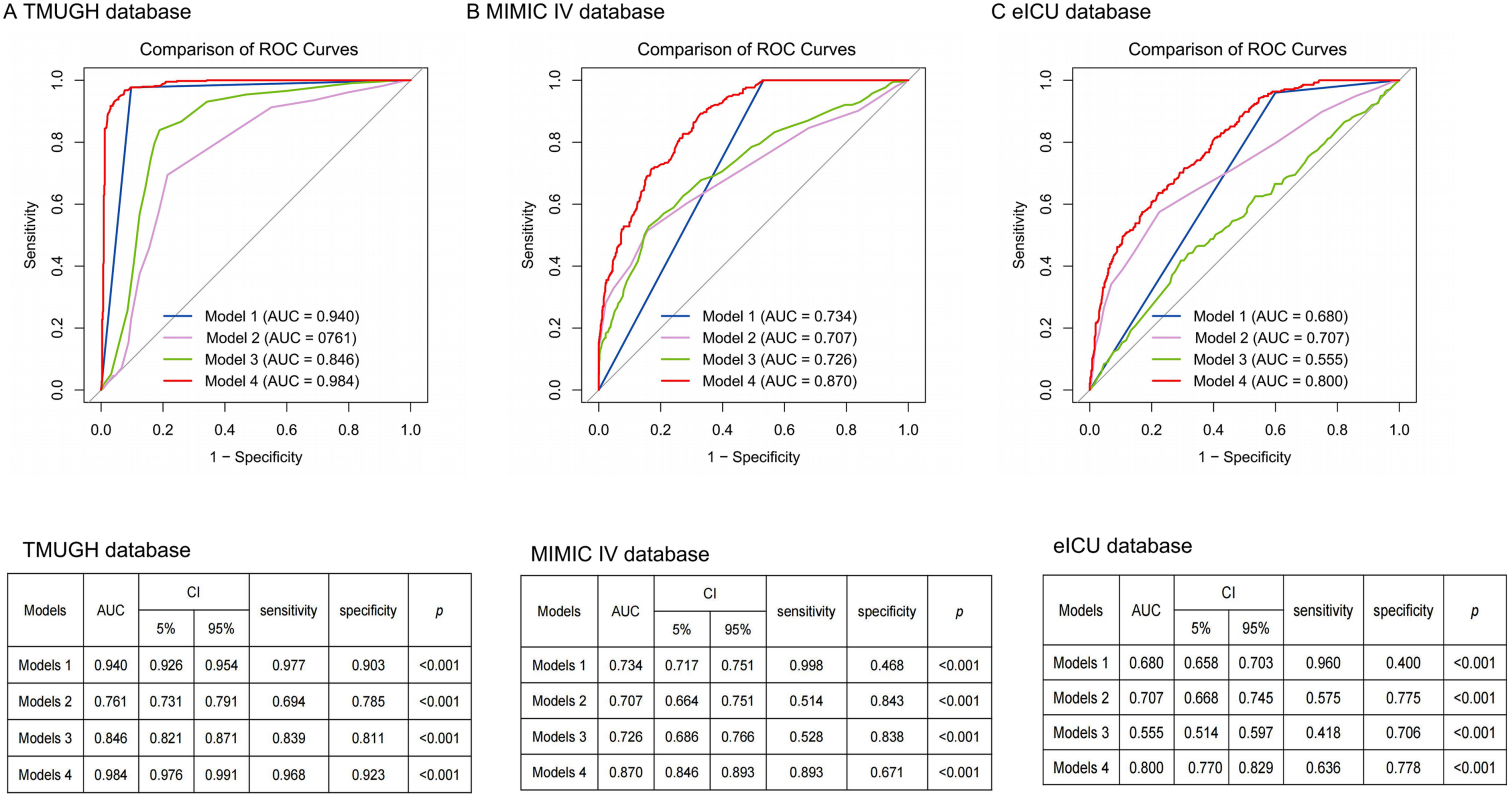
Receiver operating characteristic (ROC) curves for predicting 28-day mortality in SA-ARDS. Model 1: PNI alone; Model 2: SOFA score; Model 3: APACHE II score; Model 4: Combined model of PNI + SOFA + APACHE II. **(A)** ROC curves in the TMUGH cohort; **(B)** ROC curves in the MIMIC-IV cohort; **(C)** ROC curves in the eICU cohort. ROC, receiver operating characteristic; PNI, Prognostic Nutritional Index; SOFA, Sequential Organ Failure Assessment; APACHE II, Acute Physiology and Chronic Health Evaluation; SA-ARDS, sepsis-associated acute respiratory distress syndrome; MIMIC-IV, Medical Information Mart for Intensive Care IV.

ROC curve analysis and DeLong test were performed to assess the predictive performance of PNI, SOFA, APACHE II and their combined model (Model 4) across three cohorts, with all statistical details presented in Figure 3 and Supplementary material 5. In the TMUGH cohort, PNI (AUC = 0.940, 95%CI 0.926–0.954) was significantly superior to SOFA (AUC = 0.761, 95%CI 0.731–0.791, Z = 10.624, *p* < 0.001) and APACHE II (AUC = 0.846, 95%CI 0.821–0.871, Z = 6.342, *p* < 0.001). In the MIMIC-IV cohort, PNI (AUC = 0.734, 95%CI 0.717–0.751) showed no significant differences from SOFA (AUC = 0.707, 95%CI 0.664–0.751, Z = 1.115, *p* = 0.265) and APACHE II (AUC = 0.726, 95%CI 0.686–0.766, Z = 0.348, *p* = 0.728). In the eICU cohort, PNI (AUC = 0.680, 95%CI 0.658–0.703) was comparable to SOFA (AUC = 0.707, 95%CI 0.668–0.745, Z = −1.1703, *p* = 0.242) but outperformed APACHE II (AUC = 0.555, 95%CI 0.514–0.597, Z = 5.200, *p* < 0.001). Notably, the combined model of PNI plus SOFA and APACHE II yielded the highest AUC values in all three cohorts (TMUGH: 0.984, 95%CI 0.976–0.991; MIMIC-IV: 0.870, 95%CI 0.846–0.893; eICU: 0.800, 95%CI 0.770–0.829) and was significantly more accurate than standalone PNI in each dataset (all *p* < 0.001).

### 3.6 Association between PNI and ARDS severity

**Figure 4:**
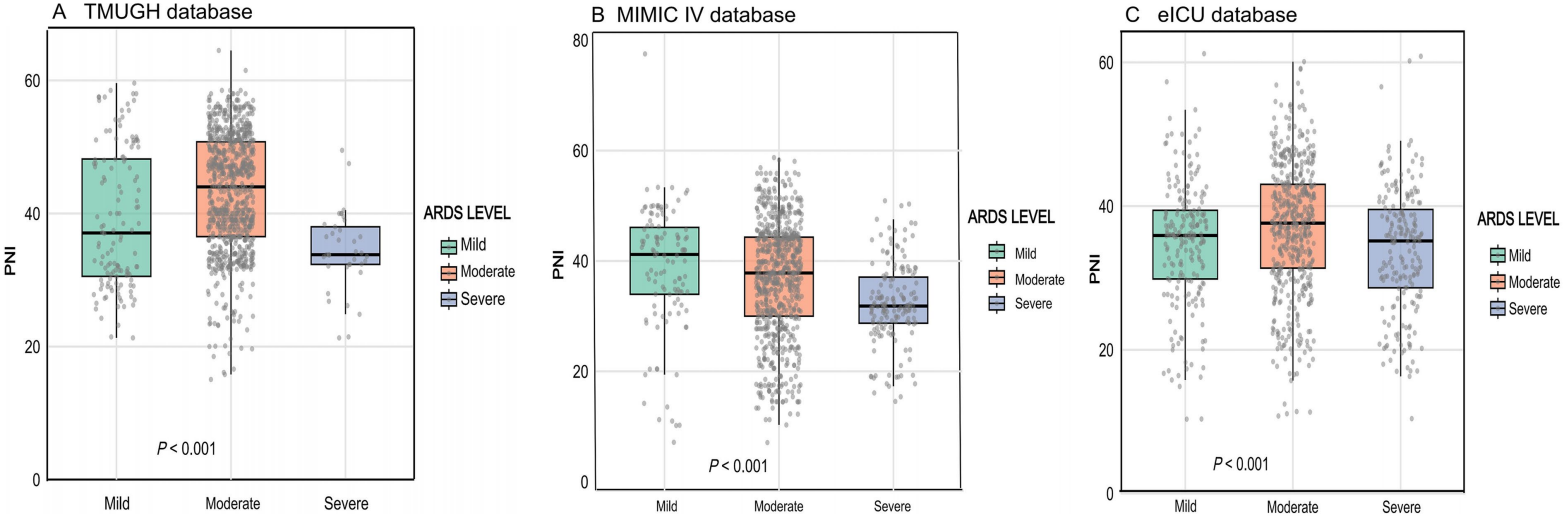
Box plots of PNI across different ARDS severity subgroups. ARDS severity was defined based on the Berlin criteria: mild (200 mmHg < PaO_2_/FiO_2_ ≤ 300 mmHg), moderate (100 mmHg < PaO_2_/FiO_2_ ≤ 200 mmHg), severe (PaO_2_/FiO_2_ ≤ 100 mmHg). **(A)** TMUGH cohort; **(B)** MIMIC-IV cohort; **(C)** eICU cohort. PNI, Prognostic Nutritional Index; ARDS, acute respiratory distress syndrome; MIMIC-IV, Medical Information Mart for Intensive Care IV; PaO_2_, arterial oxygen partial pressure; FiO_2_, fraction of inspired oxygen.

In the MIMIC IV cohort, median PNI values progressively decreased with ARDS severity: mild, moderate, and severe (Figure 4B). In the eICU and TMUGH cohorts, patients with moderate ARDS demonstrated higher median PNI values compared to those with severe and mild ARDS (Figures 4A,C).

## 4 Discussion

This multicenter cohort study demonstrates that the PNI serves as an independent protective factor against 28-day mortality in SA-ARDS patients, with a clear threshold at PNI = 41, above which the mortality risk decreased substantially. DeLong test revealed inconsistent predictive performance of standalone PNI across cohorts. In the single-center TMUGH cohort, PNI outperformed both SOFA and APACHE II. However, PNI showed comparable efficacy with the two scoring systems in the MIMIC-IV cohort, and only surpassed APACHE II in the eICU cohort. Despite such heterogeneity, the combination of PNI with SOFA and APACHE II consistently improved prognostic performance in all databases. Notably, in both the TMUGH and MIMIC-IV databases, we observed a non-linear trend between PNI levels and ARDS severity classification, with moderate ARDS patients exhibiting higher PNI values than those with mild or severe ARDS. In contrast, the eICU database revealed a progressive decline in PNI levels with increasing ARDS severity.

The varied performance of standalone PNI among the three cohorts may be explained by population differences. TMUGH is a single-center ICU cohort with relatively unified admission criteria and clinical management strategies. In contrast, MIMIC-IV and eICU are large public multicenter databases covering diverse medical institutions, with broader ranges of patient age, comorbidities, infection types and disease severity. These inherent differences in study populations and clinical settings are common in multicenter retrospective studies and may lead to heterogeneous results of a single biomarker.

Recent efforts to identify reliable prognostic biomarkers for ARDS have focused on systemic inflammatory indices, including LAR (16), NLR (17), GLR (18), and cytokine-based markers like ST2 and IL-6 (19). In contrast to these single-dimensional biomarkers, the PNI uniquely integrates nutritional (serum albumin) and immunological (lymphocyte count) parameters, offering a more holistic assessment of patient status (20). While PNI was originally developed for surgical risk stratification (21–24), its clinical utility has expanded to oncology, chronic inflammatory diseases, and severe infections (25–27). However, its prognostic value in ARDS—particularly in sepsis-associated ARDS (SA-ARDS)—remains unexplored. Our study fills this critical gap. We do not recommend using PNI alone to replace conventional SOFA or APACHE II scores. Instead, our results demonstrate that adding PNI to existing severity scoring systems yields a more powerful combined predictive model, which performs stably across different populations. Given that PNI is calculated from routine laboratory indicators and requires no complex clinical evaluation, it can be conveniently integrated into current risk assessment workflows for SA-ARDS patients.

The prognostic power of PNI is intrinsically tied to the pathophysiological mechanisms of SA-ARDS. Sepsis triggers a hyperactive systemic inflammatory response, which drives extensive lymphocyte apoptosis and immune cell exhaustion (28). Progressive lymphopenia impairs host immune surveillance and pathogen clearance, further amplifying inflammatory cytokine release and aggravating alveolar-capillary barrier damage, a hallmark lesion of SA-ARDS. Serum albumin, another constituent of PNI, exerts multiple protective effects against inflammation-induced lung injury: it suppresses pro-inflammatory mediators, neutralizes reactive oxygen species, stabilizes pulmonary endothelium and reduces vascular permeability, thereby alleviating inflammatory pulmonary edema (29, 30). As a combined marker of peripheral lymphocyte count and serum albumin, PNI simultaneously reflects immune function, nutritional status and systemic inflammatory load. This dual biological basis enables PNI to effectively predict 28-day mortality in SA-ARDS patients. PNI is derived from routine laboratory tests and can be readily applied clinically. While its standalone predictive ability differed across cohorts, combining PNI with SOFA and APACHE II consistently achieved better prognostic performance. Given that this study only demonstrates an associative relationship, further interventional research is needed to verify whether optimizing PNI-related indicators can improve patient outcomes.

PNI transcends the limitations of unidimensional biomarkers by integrating two ARDS-relevant parameters, thereby minimizing confounding from isolated abnormalities. Importantly, PNI reflects the bidirectional relationship between nutrition and immunity—malnutrition impairs immune responses, while immune activation exacerbates metabolic depletion—a cycle pivotal in ARDS progression. Clinically, PNI’s simplicity is transformative: unlike SOFA or APACHE II, which require complex clinical data, PNI relies solely on routine laboratory tests, making it feasible even in resource-limited settings. Combined with ROC curve analysis and DeLong test results, PNI exhibits reliable discriminatory power for 28-day mortality. The predictive ability of standalone PNI varied across cohorts, so we cannot draw a universal conclusion that PNI is superior to SOFA or APACHE II. Nevertheless, the complementary effect between PNI and traditional scoring systems was consistent: the combined model always achieved the best discriminative ability for 28-day mortality. This complementary value is the main clinical advantage of PNI in SA-ARDS risk stratification. Combined with its simple calculation, PNI can act as a potential alternative biomarker for rapid, cost-effective risk assessment in clinical settings. With simple calculation and reliable prognostic performance, PNI can act as a potential alternative biomarker for rapid, cost-effective risk assessment in clinical settings. Since our study only reveals an association between PNI and mortality rather than causality, and relevant interventional evidence is insufficient, we do not currently recommend using PNI to guide therapeutic decisions. Future prospective and interventional studies are warranted to explore the clinical value of PNI-guided individualized management for ARDS patients.

In line with the GAM results, we identified a clear threshold at a PNI value of 41, which serves as a clinically actionable cutoff for risk stratification. Patients with PNI values below this threshold presented significantly higher mortality risk, which suggests that this population may require close clinical monitoring. Whether targeted interventions aiming to improve PNI can improve patient outcomes remains to be verified by interventional studies. Whether therapeutic strategies specifically targeting improvements in PNI components—such as nutritional supplementation or immunomodulatory approaches—could modify outcomes in ARDS remains an important question for future interventional studies. The complex relationship between PNI and ARDS severity classifications observed in our study merits further investigation. However, we can observe that across all three databases, PNI levels gradually declined as ARDS severity increased among patients with moderate and severe ARDS. As the severity of ARDS increases, the PNI level gradually decreases, indicating the importance of PNI in assessing severe ARDS patients.

We further analyzed the correlation between PNI and ARDS severity stratified by the Berlin criteria, and observed inconsistent trends across the three datasets, which can be reasonably explained by database attributes and the baseline clinical characteristics of enrolled patients in our study. TMUGH and MIMIC-IV are two independent single-center cohorts with unified diagnostic criteria and treatment protocols for SA-ARDS, but they yielded different relationships between PNI and ARDS severity. In the MIMIC-IV cohort, median PNI decreased progressively along with the increase of ARDS severity, showing an obvious linear negative correlation. Combined with the baseline data of this cohort, patients with more severe ARDS had higher levels of inflammatory biomarkers, more severe organ dysfunction and metabolic disorders. These pathological changes continuously reduced serum albumin and peripheral lymphocyte counts, resulting in a steady drop of PNI as ARDS aggravated. In the TMUGH single-center cohort, however, PNI distribution presented a non-linear pattern: moderate ARDS patients had higher median PNI than patients with mild and severe ARDS. As shown in the baseline comparisons of laboratory and clinical parameters, mild ARDS patients in this cohort already had relatively low albumin and lymphocyte levels at admission. Meanwhile, severe ARDS patients were complicated with overwhelming systemic inflammation and multiple organ failure, which also caused a significant reduction in PNI. Consequently, moderate ARDS patients, with relatively intact nutritional and immune indicators, had the highest PNI values in this cohort. Unlike the above two single-center databases, eICU is a multicenter collaborative dataset covering numerous medical facilities. Our baseline analyses demonstrated that the eICU cohort had wider variations in age, comorbidity composition, pathogen distribution and supportive treatment strategies compared with single-center populations. The diversified patient profiles and clinical practices across different centers contributed to a different correlation pattern. Similar to the TMUGH cohort, moderate ARDS patients in eICU also had higher PNI than mild and severe cases.

Importantly, one consistent trend existed across all three cohorts: PNI decreased continuously from moderate to severe ARDS. Combined with the results of regression analysis, survival analysis and ROC analysis, PNI was confirmed as an independent protective factor for 28-day mortality in SA-ARDS patients in all datasets. Even though the association between PNI and mild ARDS varied by cohort, PNI still performs stably for evaluating disease progression in moderate and severe SA-ARDS, the high-risk population in clinical practice.

The current study has several distinct novelties. First, this is the first multicenter study to systematically explore the prognostic value of PNI in SA-ARDS patients and validate its predictive performance across three independent ICU cohorts. Second, we identified an optimal PNI cutoff value of 41 for 28-day mortality risk stratification, and confirmed its stable threshold effect via multiple statistical methods. Third, we verified that combining PNI with classic critical care scoring systems (SOFA, APACHE II) can significantly improve prognostic efficiency across different populations, providing a simple, low-cost auxiliary risk stratification strategy for clinical practice. Fourth, we revealed heterogeneous correlations between PNI and ARDS severity across different databases, which provides new clues for subsequent stratified research on SA-ARDS.

Certainly, our study still has multiple limitations that need to be acknowledged. First, this is a retrospective observational study. Although we adopted multiple statistical methods including PSM, IPW and doubly robust estimation to control known confounding factors, unmeasured variables such as long-term medication history, subtle differences in infection progression and individual genetic background may still interfere with the results. More importantly, retrospective design can only demonstrate an associative relationship rather than causal inference between PNI and 28-day mortality. Second, we only collected single-point baseline PNI values measured within 24 h after ICU admission, and lacked continuous dynamic monitoring of PNI during the entire ICU stay. The dynamic changing trends of PNI in survivors and non-survivors, as well as the prognostic value of PNI variation over time, remain unclear. Whether continuous decline or insufficient recovery of PNI during hospitalization can predict poor outcomes needs further prospective research with serial laboratory tests. Third, all enrolled patients met the criteria for invasive mechanical ventilation. Therefore, our conclusions are only applicable to SA-ARDS patients receiving mechanical ventilation, and cannot be extended to patients with mild SA-ARDS who do not require ventilator support. Fourth, we did not perform further subgroup analyses stratified by infection pathogens, types of underlying diseases, or major supportive treatments such as prone positioning ventilation and corticosteroid therapy. It remains unknown whether the prognostic efficacy of PNI differs in these distinct subgroups. Fifth, this study did not explore the interaction between PNI changes and clinical treatment adjustment. Whether nutritional support, immunomodulatory therapy or albumin supplementation can improve PNI levels and further reverse adverse clinical outcomes still lacks corresponding interventional evidence (31–34). In view of the above limitations, large-sample, prospective cohort studies with dynamic PNI monitoring, as well as targeted interventional trials, are urgently required in the future to validate our findings and explore the clinical application value of PNI-guided individualized management strategies for SA-ARDS patients.

## 5 Conclusion

This multicenter retrospective study confirms that PNI is an independent prognostic marker for 28-day mortality in patients with SA-ARDS, with a validated optimal cutoff value of 41. The predictive performance of standalone PNI differed across the single-center TMUGH cohort and two public databases (MIMIC-IV, eICU), which may be related to differences in study populations and clinical backgrounds. Notably, combining PNI with SOFA and APACHE II significantly improved prognostic accuracy in all three cohorts, indicating that PNI can serve as an effective auxiliary indicator to complement traditional severity scores. Since PNI relies on routine blood tests and is easy to calculate, it is suitable for widespread clinical application. Of note, this observational study only demonstrates an associative relationship rather than causality. At present, interventional studies targeting PNI are still lacking. Further prospective clinical trials are needed to verify whether PNI-guided strategies can improve clinical outcomes of SA-ARDS patients.

## Data Availability

Publicly available datasets were analyzed in this study. This data can be found here: the MIMIC IV database (version 1.0) is publicly available at: https://mimic-iv.mit.edu/ and the eICU database is publicly available at: https://eicu-crd.mit.edu/about/eicu/. Any studyer who adheres to the data use requirements is permitted access to these databases.

## Glossary

ALT: Alanine Aminotransferase
APTT: Activated Partial Thromboplastin Time
APACHE II: Acute Physiology and Chronic Health Evaluation II
ARDS: Acute Respiratory Distress Syndrome
CK: Creatine Kinase
CK-MB: Creatine Kinase-MB
CI: Confidence Interval
CRRT: Continuous Renal Replacement Therapy
CRP: C-Reactive Protein
FiO_2_: Fraction of Inspired Oxygen
GAM: Generalized Additive Models
ICU: Intensive Care Unit
IPW: Inverse Probability Weighting
INR: International Normalized Ratio
KM: Kaplan–Meier
Lac: Lactate
MIMIC-IV: Medical Information Mart for Intensive Care IV
MV: Mechanical Ventilation
OR: Odds Ratio
PaCO_2_: Partial pressure of arterial carbon dioxide
PaO_2_: Partial pressure of arterial oxygen
PCT: Procalcitonin
PEEP: Positive End-Expiratory Pressure
PNI: Prognostic Nutritional Index
PSM: Propensity Score Matching
PT: Prothrombin Time
ROC: Receiver Operating Characteristic;
RR: Respiratory Rate
SA-ARDS: Sepsis-associated Acute Respiratory Distress Syndrome
SBP: Systolic Blood Pressure
DBP: Diastolic Blood Pressure
SOFA: Sequential Organ Failure Assessment
SpO_2_: Peripheral Oxygen Saturation
TV.kg. IBWT: Tidal volume per kilogram of ideal body weight
WBC: White Blood Cell count
