# Supplementary material for: Association between prognostic nutritional index and 28-day mortality in patients with sepsis-associated acute respiratory distress syndrome
Source: Front Nutr. 2026 Jul 7;13:1867675. doi: 10.3389/fnut.2026.1867675 (PMC13384935; doi:10.3389/fnut.2026.1867675)
Supplement: Supplementary file 1 [file Table_1.DOCX]

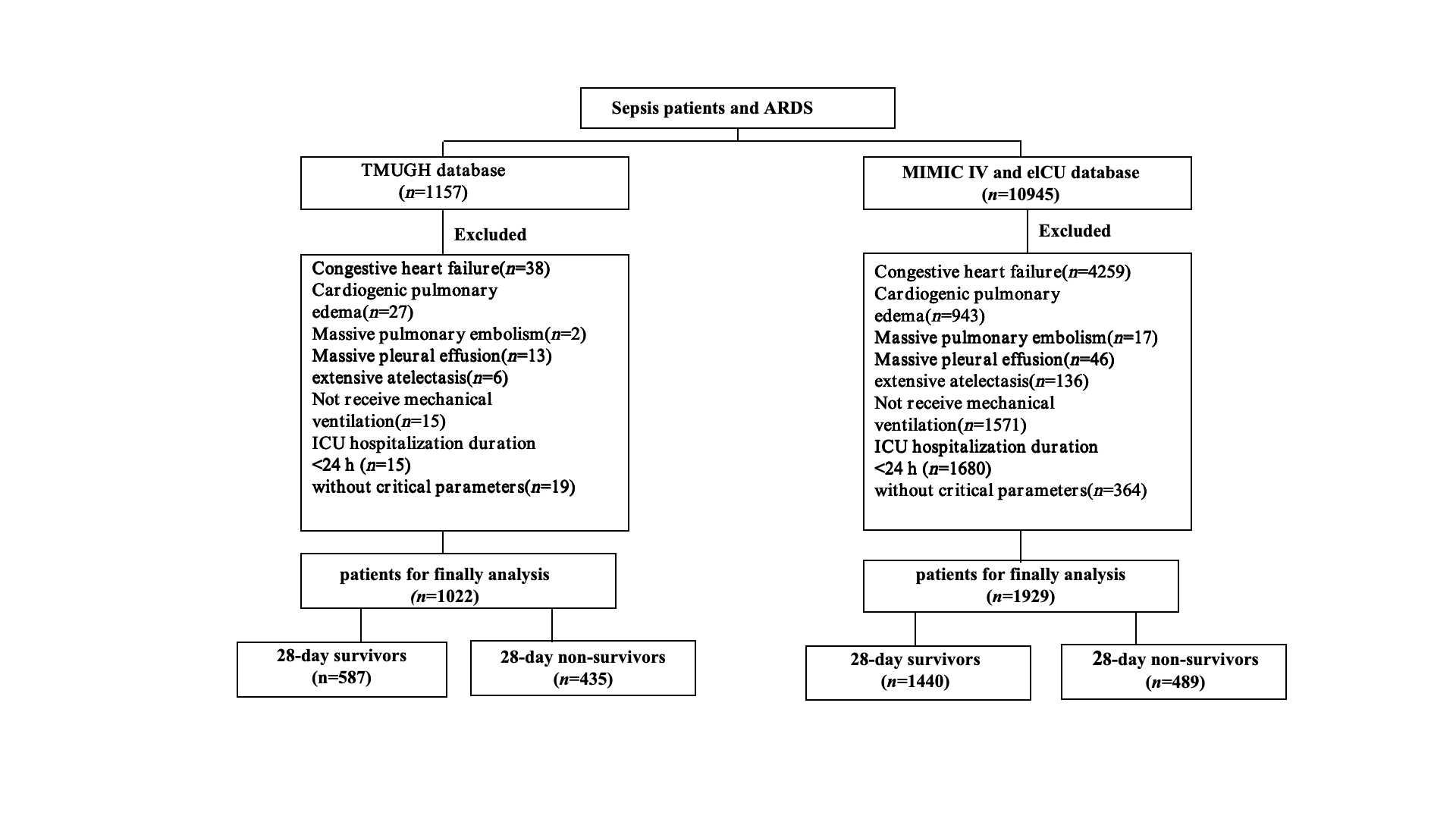


Supplementary Material 1: Flow chart for patient selection

Abbreviations: ARDS : Acute Respiratory Distress Syndrome; MIMIC-IV : Medical Information Mart for Intensive Care IV; TMUGH : Tianjin Medical University General Hospital; eICU : eICU Collaborative Research Database.

**Supplementary Material 2:** Baseline characteristics of patients with sepsis-associated acute respiratory distress syndrome (SA-ARDS)

|  | Original cohort | | | Matched cohort | | |
| --- | --- | --- | --- | --- | --- | --- |
|  | Survival group  (*n* : 587) | Non-survival group (*n* : 435) | *P* | Survival group  (*n* : 435) | Non-survival group(*n* : 435) | *P* |
| **Vital signs,(median[IQR])** | | | | | | |
| Temperature | 37.60[36.70,  38.30] | 37.60[36.60,  38.30] | 0.591 | 37.50 [36.70, 38.30] | 37.50 [36.50, 38.20] | 0.102 |
| SBP(mmHg) | 119.00[100.50,143.00] | 114.00[88.00  ,140.00] | 0.002 | 118.00 [98.00, 141.75] | 114.00 [85.00, 140.00] | 0.057 |
| DBP(mmHg) | 73.00[51.00  ,84.00] | 70.00[45.00  ,83.00] | 0.011 | 72.00 [50.00, 84.75] | 70.00 [42.00, 85.00] | 0.06 |
| HR(bpm) | 104.00[87.00  ,122.00] | 110.00[88.50  ,125.00] | 0.059 | 105.00 [88.25, 122.00] | 109.00 [88.00, 124.00] | 0.410 |
| **Respiratory-related indicators, (median [IQR])** | | | | | | |
| PaCO_2_(mmHg) | 38.00[31.80,  42.80] | 38.00[32.00,  44.10] | 0.683 | 37.80 [30.80, 42.00] | 38.10 [32.80, 44.40] | 0.076 |
| Breathing Rate(bpm) | 22.50[17.50,  29.30] | 22.10[16.80,  29.00] | 0.244 | 22.45 [17.50, 29.30] | 22.50 [16.80, 30.70] | 0.927 |
| **Microbiology type, (*n* (%))** | | | | | | |
| *Acinetobacter*  *baumannii* | 89(15.2) | 112(25.7) | <0.001 | 79(20.1) | 74(18.8) | 0.719 |
| *Escherichia coli* | 55(9.4) | 30(6.9) | 0.193 | 41(10.4) | 12(3.0) | <0.001 |
| *Klebsiella*  *pneumoniae* | 112(19.1) | 134(30.8) | <0.001 | 86(21.8) | 84(21.3) | 0.931 |
| *Staphylococcus aureus* | 61(10.4) | 51(11.7) | 0.567 | 40(10.2) | 42(10.7) | 0.907 |
| *Pseudomonas aeruginosa* | 80(13.6) | 40(9.2) | 0.038 | 65(16.5) | 40(10.2) | 0.012 |
| **Laboratory parameters，(median[IQR])** | | | | | | |
| WBC(×10^9^/L) | 13.32[9.52,  19.40] | 14.08[9.31,  20.28] | 0.542 | 12.98 [9.41, 19.39] | 14.10 [9.38, 20.89] | 0.278 |
| Hemoglobin  (g/L) | 88.00[78.00,  108.00] | 85.00[74.00,  109.00] | 0.079 | 88.00[78.00,  108.00] | 88.00[78.00,  111.00] | 0.798 |
| ALT (U/L) | 32.00[18.00,  75.00] | 34.00[20.00,  87.50] | 0.150 | 35.00[20.00,  82.00] | 31.00[18.00,  84.00] | 0.250 |
| AST (U/L) | 48.00[30.00,  96.00] | 59.00[32.50,  131.50] | 0.001 | 52.00 [30.00, 110.00] | 55.00 [31.00, 112.00] | 0.260 |
| Total Bilirubin  (μmol/L) | 14.30[9.30,  24.15] | 17.40[10.25,  28.25] | 0.011 | 15.00 [9.43, 27.87] | 17.40 [10.30, 23.70] | 0.374 |
| CK (U/L) | 222.00[87.50,705.50] | 282.00[97.50,896.00] | 0.009 | 239.50 [92.00, 847.00] | 221.00 [90.00, 841.00] | 0.737 |
| CKMB (U/L) | 22.00[12.00,  51.00] | 29.00[14.50,  90.00] | <0.001 | 27.00 [13.00, 80.75] | 29.00 [13.00, 80.00] | 0.299 |
| Cr (μmol/L) | 138.00[81.00,287.00] | 182.00[89.50,319.50] | 0.004 | 149.50 [81.00, 299.50] | 153.00 [89.00, 287.00] | 0.531 |
| BUN (mg/dL) | 14.10[8.35,  21.00] | 17.00[9.00,  23.00] | 0.021 | 15.00[8.93,  22.17] | 15.00[9.00,  21.00] | 0.665 |
| PT (s) | 13.10[11.90,  14.80] | 13.80[12.10,  17.30] | <0.001 | 13.20[12.00,  15.10] | 13.70[12.00,  15.30] | 0.261 |
| INR | 1.20  [1.09,1.34] | 1.27  [1.11,1.47] | 0.001 | 1.24  [1.10,1.38] | 1.24  [1.09,1.40] | 0.802 |
| APTT (s) | 30.80[28.20,  34.40] | 31.70[28.60,  38.00] | 0.008 | 31.10 [28.30, 34.90] | 31.10 [28.40, 38.20] | 0.183 |
| Fibrinogen (g/L) | 3.82[2.88,  4.87] | 3.77[2.71,  4.74] | 0.076 | 3.76[2.73,  4.86] | 3.84[2.85,  4.74] | 0.438 |
| CRP (mg/dL) | 10.85[3.99,  20.00] | 17.30[5.50,  20.00] | 0.001 | 11.85[4.07,  20.00] | 15.10[5.18,  20.00] | 0.119 |
| PCT (ng/mL) | 2.38[0.80,  8.93] | 3.19[1.11,  10.95] | 0.012 | 2.66[0.89,  9.29] | 3.07[1.18,  14.55] | 0.148 |
| Glu (mmol/L) | 12.00[8.80,18.00] | 11.90[8.70,18.10] | 0.586 | 12.30[8.80,  18.30] | 11.70[8.80,  17.70] | 0.268 |
| PLT (×10^9^/L) | 134.00[81.00,209.00] | 103.00[43.50,180.50] | <0.001 | 118.00[70.00,  193.00] | 128.00[78.00,  189.00] | 0.668 |
| Lac-0 hour(mmol/L) | 1.80  [1.30,2.90] | 2.40  [1.50,4.90] | <0.001 | 1.90  [1.40,3.48] | 2.20  [1.50,4.00] | 0.035 |
| Lac-6 hour (mmol/L) | 1.80  [1.30,2.80] | 2.40  [1.70,4.30] | <0.001 | 1.90[1.40,3.08] | 2.20[1.50,3.80] | 0.013 |
| Lac-12 hour(mmol/L) | 1.80  [1.30,2.80] | 2.40  [1.50,3.80] | <0.001 | 1.80  [1.30,3.00] | 2.20  [1.70,3.80] | <0.001 |
| Lac-18 hour (mmol/L) | 1.70  [1.30,2.40] | 2.20  [1.60,3.30] | <0.001 | 1.80  [1.30,2.50] | 2.20  [1.50,3.00] | <0.001 |
| Lac-24 hour(mmol/L) | 1.70  [1.25,2.30] | 2.20  [1.50,3.20] | <0.001 | 1.80  [1.30,2.50] | 2.00  [1.40,3.10] | <0.001 |
| **Comorbidities, (*n*(%))** | | | | | | |
| Hypertension | 322(54.9) | 239(54.9) | 1.000 | 216(54.8) | 212(53.8) | 0.83 |
| Diabetes Mellitus | 196(33.4) | 124(28.5) | 0.110 | 135(34.3) | 104(26.4) | 0.02 |
| Coronary Artery Disease | 186(31.7) | 137(31.5) | 1.000 | 119(30.2) | 116(29.4) | 0.876 |
| Chronic Kidney Disease | 132(22.5) | 108(24.8) | 0.425 | 94(23.9) | 70(17.8) | 0.044 |
| Cancer | 91(15.5) | 101(23.2) | 0.002 | 56(14.2) | 70(17.8) | 0.206 |
| Cirrhosis | 75(12.8) | 67(15.4) | 0.268 | 52(13.2) | 48(12.2) | 0.748 |
| Immunosuppression | 71(12.1) | 58(13.3) | 0.621 | 52(13.2) | 52(13.2) | 1.000 |
| **Site of infection，(*n*(%))** | | | | | | |
| Lung | 512(87.2) | 377(86.7) | 0.867 | 352(89.3) | 332(84.3) | 0.046 |
| Abdominal | 132(22.5) | 83(19.1) | 0.214 | 93(23.6) | 66(16.8) | 0.021 |
| Urinary | 54(9.2) | 23(5.3) | 0.026 | 40(10.2) | 24(6.1) | 0.05 |
| Skin and soft tissue | 24(4.1) | 15(3.4) | 0.716 | 17(4.3) | 18(4.6) | 1.000 |
| Catheter | 29(4.9) | 26(6.0) | 0.558 | 20(5.1) | 18(4.6) | 0.868 |
| **The score system (median[IQR])** | | | | | | |
| SOFA | 8.00  [5.00,8.00] | 10.00  [8.00,12.00] | <0.001 | 8.00  [7.00,10.00] | 9.00  [8.00,10.00] | <0.001 |
| APACHE II | 14.00[13.00,  19.00] | 24.00[21.00,  27.00] | <0.001 | 15.00[13.00,  20.00] | 24.00[21.00,  25.00] | <0.001 |
| **The outcome of sepsis-associated ARDS patients** | | | | | | |
| Length of hospital stay  (median[IQR]) | 15.00[8.63,28.73] | 9.91[4.26,  18.00] | <0.001 | 18.48[10.82,  33.94] | 7.35[2.95,  12.38] | <0.001 |

SBP: Systolic blood pressure; DBP: Diastolic blood pressure; HR: Heart rate; PaCO₂: Partial pressure of arterial carbon dioxide; WBC: White blood cell count; ALT: Alanine aminotransferase; AST: Aspartate aminotransferase; CK: Creatine kinase; CK-MB: Creatine kinase-MB isoenzyme; Cr: Creatinine; BUN: Blood urea nitrogen; PT: Prothrombin time; INR: International normalized ratio; APTT: Activated partial thromboplastin time; CRP: C-reactive protein; PCT: Procalcitonin; PLT: Platelet count; Lac: Lactate; SOFA: Sequential Organ Failure Assessment; APACHE II: Acute Physiology and Chronic Health Evaluation II.

**
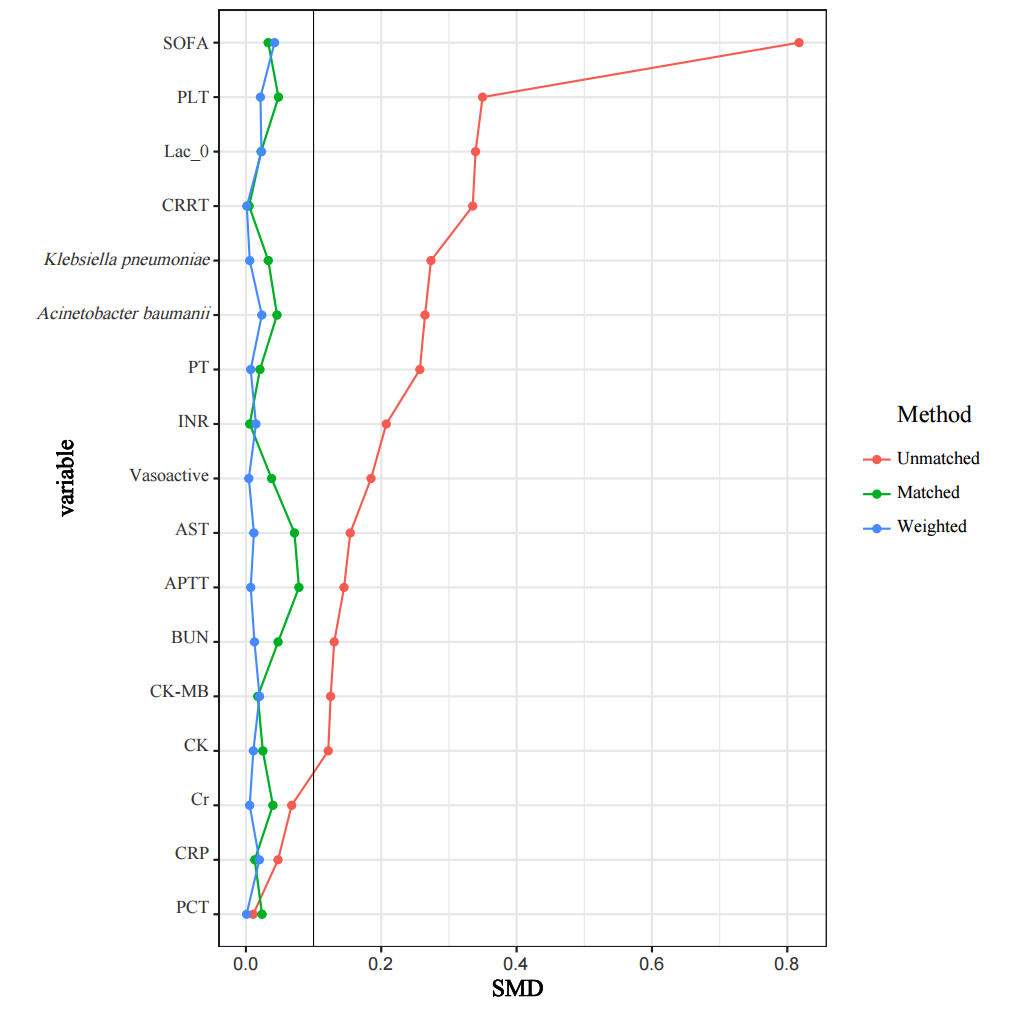
**

Supplementary Material 3: Propensity score (PSM) analysis of risk factors for 28-day mortality in patients with SA-ARDS. Abbreviations: PSM : Propensity Score Matching; SMD : Standardized Mean Difference; SOFA : Sequential Organ Failure Assessment; PLT : Platelet count; Lac : Lactate; CRRT : Continuous Renal Replacement Therapy; PT : Prothrombin Time; INR : International Normalized Ratio; AST : Aspartate Aminotransferase; APTT : Activated Partial Thromboplastin Time; BUN : Blood Urea Nitrogen; CK-MB : Creatine Kinase-MB; CK : Creatine Kinase; Cr : Creatinine; CRP : C-Reactive Protein; PCT : Procalcitonin..

**Supplementary Material 4:** Multivariate logistic regression analysis of risk factors for 28-day mortality in patients with SA-ARDS

| **Variable** | ***P*** | **OR** | **95%CI** | |
| --- | --- | --- | --- | --- |
| SpO_2_ | 0.002 | 0.47 | 0.28 | 0.73 |
| PaO_2_/FiO_2_ | 0.175 | 1.05 | 0.98 | 1.13 |
| Duration of MV | 0.211 | 1.07 | 0.97 | 1.2 |
| TV.kg.IBW | 0.981 | 0.99 | 0.58 | 1.68 |
| PaO_2_ | 0.149 | 1.05 | 0.98 | 1.13 |
| PEEP | 0.056 | 1.93 | 0.99 | 3.82 |
| SOFA | 0.538 | 1.07 | 0.87 | 1.32 |
| Cr | 0.096 | 1.00 | 0.99 | 1.00 |
| BUN | 0.862 | 1.01 | 0.94 | 1.07 |
| PT | 0.444 | 1.08 | 0.88 | 1.29 |
| APTT | 0.047 | 1.03 | 0.99 | 1.06 |
| CRP | 0.737 | 1.01 | 0.97 | 1.03 |
| PCT | 0.496 | 0.99 | 0.96 | 1.02 |
| Lac | 0.785 | 0.98 | 0.81 | 1.16 |
| APACHE Ⅱ | <0.001 | 1.37 | 1.19 | 1.62 |
| Length of hospital stay | 0.028 | 0.88 | 0.79 | 0.97 |
| PNI | <0.001 | 0.41 | 0.31 | 0.51 |
| SBP | 0.244 | 1.01 | 0.99 | 1.03 |
| DBP | 0.239 | 0.98 | 0.94 | 1.01 |
| PLT | 0.747 | 1.00 | 0.99 | 1.00 |
| AST | 0.394 | 1.00 | 1.00 | 1.00 |
| Total Bilirubin | 0.007 | 0.99 | 0.98 | 1.00 |
| CK | 0.357 | 1.00 | 1.00 | 1.00 |
| CK-MB | 0.779 | 1.00 | 1.00 | 1.00 |

SBP: systolic blood pressure; DBP: diastolic blood pressure; AST: aspartate aminotransferase; CK: creatine kinase; CKMB: creatine kinase-myocardial band; Cr: creatinine; BUN: blood urea nitrogen; PT: prothrombin time; INR: international normalized ratio; APTT: activated partial thromboplastin time; CRP: C-reactive protein; PCT: procalcitonin; PLT: platelet count; Lac: lactate; SOFA: Sequential Organ Failure Assessment; APACHE II: Acute Physiology and Chronic Health Evaluation II; SpO₂: saturation of peripheral oxygen; PaO₂: partial pressure of arterial oxygen; FiO₂: fraction of inspired oxygen; PEEP: positive end-expiratory pressure; PNI: prognostic nutritional index (10 × albumin [g/dL] + 5 × lymphocyte count [×10^9^/L]); CRRT: continuous renal replacement therapy; MV: mechanical ventilation; TV/kg IBW: tidal volume per kilogram of ideal body weight. Statistical significance was defined as *P* < 0.05.

**Supplementary Material 5**: Detailed results of the DeLong test for pairwise comparison of ROC curves

| **TMUGH cohort** | | |
| --- | --- | --- |
|  | *Z-value* | *P*-value |
| Model 1 vs Model 2 | 10.624 | < 0.001 |
| Model 1 vs Model 3 | 6.342 | < 0.001 |
| Model 1 vs Model 4 | -7.585 | < 0.001 |
| **MIMIC IV cohort** | | |
| Model 1 vs Model 2 | 1.115 | 0.265 |
| Model 1 vs Model 3 | 0.349 | 0.728 |
| Model 1 vs Model 4 | -11.367 | < 0.001 |
| **eICU cohort** | | |
| Model 1 vs Model 2 | -1.170 | 0.242 |
| Model 1 vs Model 3 | 5.200 | < 0.001 |
| Model 1 vs Model 4 | -9.287 | < 0.001 |

MIMIC-IV: Medical Information Mart for Intensive Care IV; TMUGH: Tianjin Medical University General Hospital; eICU: eICU Collaborative Research Database. Model 1: PNI; Model 2: SOFA; Model 3: APACHE II; Model 4: PNI+SOFA+APACHE II.
